# A novel insertion design of fiber materials for the adhesive reattachment in vertically fractured teeth

**DOI:** 10.1371/journal.pone.0258534

**Published:** 2021-10-13

**Authors:** Safa Kurnaz, Ayşe Diljin Keçeci

**Affiliations:** 1 Faculty of Dentistry, Department of Endodontics, Kutahya Health Sciences University, Kutahya, Turkey; 2 Faculty of Dentistry, Department of Endodontics, Suleyman Demirel University, Isparta, Turkey; Virginia Commonwealth University, UNITED STATES

## Abstract

**Objective:**

This *ex vivo* study aimed to evaluate the strengthening effect of different ferrule and reattachment designs with fiber and adhesive materials on vertically fractured teeth.

**Methods:**

Ninety extracted single-root premolars were instrumented and divided into nine groups (two control groups and seven experimental groups; n = 10). The negative control (NC) group comprised of intact teeth, while the positive control (PC) group comprised of root canal-treated teeth. The roots of the teeth in the experimental groups were vertically fractured into two equal fragments. The fragments were reattached with one of the followings: 4-methacryloxyethyl trimellitate anhydride/methacrylate-tri-n-butyl borane (4-META/MMA-TBB) resin, 4-META/MMA-TBB + quartz fiber post, 4-META/MMA-TBB + glass fiber bundles, 4-META/MMA-TBB + quartz fiber post + 1 mm ferrule, 4-META/MMA-TBB + glass fiber bundles + 1 mm ferrule, 4-META/MMA-TBB + quartz fiber post + 2 mm ferrule, and 4-META/MMA-TBB + glass fiber bundles + 2 mm ferrule. The core build-ups were made with composite resin. The specimens were subjected to compressive loading until failure occurred. Mean load necessary to fracture each sample and the fracture types of these samples were recorded.

**Results:**

The highest mean fracture load was recorded in the NC group (1,036.7 N), which was not significantly higher than the PC group (989.66 N) (p > 0.05). The roots reattached with quartz fiber post demonstrated significantly less fracture strength (871.9 N) as compared to the other test and control groups (p < 0.05). There was no significant difference between the PC group and reattached fragments with different ferrule designs in terms of fracture resistance (p > 0.05).

**Conclusions:**

The customized fiber bundles may be more suitable for reattachment of vertically fractured teeth than the rigid fiber posts. For reattachment procedures, the ferrule design may be preferred to increase the fracture strength of vertically fractured teeth.

## Introduction

Vertical root fracture (VRF) constitutes a longitudinal fracture of the root occurring at any level, usually directed buccolingually [1]. Rivera and Walton [2] described the following five types of longitudinal fractures: enamel craze lines, fractured cusps, cracked tooth, split tooth, and VRF. The VRF terminology has recently been changed to “root originated fracture” [3]. The reported prevalence of VRF is 4.4–10.6% among extracted teeth [4,5] and 11–30% in endodontically treated teeth [6,7]. The etiology of VRF is multifactorial and related to several predisposing and iatrogenic factors including tooth morphology, loss of moisture in a pulpless tooth, cracks on the tooth, loss of hard tissue due to extensive caries and trauma, parafunctional habits, cracked tooth syndrome, excessive occlusal forces, acute trauma, overpreparation, and overinstrumentation of root canals [1,8,9]. VRF can weaken the tooth to the point that it cannot withstand normal masticatory forces and is susceptible to fracture, thus complicating the restorative process [10]. Curved and narrow roots are most susceptible to VRF; these include the roots of maxillary second premolars, mandibular incisors, mandibular premolars, and mandibular molars and the mesiobuccal roots of maxillary molars [11].

The debate on whether a compromised fractured tooth should be preserved by alternative treatments or extracted and substituted with an implant-supported restoration has not been resolved [12–14]. However, it is observed that implants may be associated with more complications and may require more postoperative care as compared to the natural tooth; hence, the argument may swing in favor of endodontics and tooth preservation [13,14].

With the advantages of modern endodontics, it is possible to treat various VRF cases [15–20]. Cone beam computed tomography has proved more helpful than periapical radiographs in diagnosing and monitoring VRF [20]. Other technical supports such as magnification and illumination for microsurgery and the development of adhesive materials offer alternatives to tooth extraction [9,13]. The strengthening of vertically fractured teeth using adhesive systems and/or fiber materials has also been examined in *ex vivo* studies [21,22]. Reattachment can be improved using adhesive systems and fiber materials. Adhesive systems include flowable resin materials, 4-methacryloxyethyl trimellitate anhydride/methacrylate-tri-n-butyl borane (4-META/MMA-TBB), and dual-cure cements. Fiber materials include posts (e.g., carbon, quartz, and glass) with flexible fibers (e.g., polyethylene and glass) in different directions (woven fibers [Ribbond, Seattle, WA, USA] and mesh fibers [Everstick NET, Stick Tech Ltd., Turku, Finland]) and unidirectional fibers (Everstick, Stick Tech Ltd., Turku, Finland) [16,17,19,21].

The effect of ferrule designs using fibers on the reattachment of vertically fractured teeth has not been investigated previously. This study aimed to evaluate the compressive strength of vertically fractured teeth that were reattached using 4-META/MMA-TBB resin-based cement and/or quartz fiber posts, as well as glass fiber bundles, in different insertion manners such as the root canal or around the cervical area.

Two hypotheses were tested: first, there was no significant difference in fracture strength or fracture types of reattached fragments between the rigid fibers and flexible fiber bundles; and second, a ferrule design with fiber bundles did not increase the fracture strength of the reattached teeth.

## Materials and methods

Single straight-rooted mandibular premolars extracted for orthodontic or periodontal reasons with approximately same dimensions were selected and stored in distilled water until use. They were used following informed consent from patients and approval of the research protocol by the Ethical Committee of the Medical Faculty of Suleyman Demirel University (protocol number: 2014–31). Written consent was given, all patients signed informed consent forms before enrollment, and only adult teeth were used for this study. The mean mesiodistal and buccolingual dimensions were 6.0 mm and 7.5 mm, respectively, and the mean length was 22 mm. Roots with less than ±10% variation from these values were used. A periodontal scaler was used to remove calculus and soft tissues from the root surfaces. All selected teeth were without caries, restorations, fractures, or cracks. Curved, internally or externally resorbed, and calcified canals were excluded following radiographic examination. The buccolingual and mesiodistal dimensions were measured at the cementoenamel junction (CEJ). In the experimental groups, each tooth was decoronated using a diamond disc under a water coolant at the CEJ to obtain standard root lengths of 16 mm.

The negative control (NC) group was composed of sound teeth without root canal treatment. In the positive control (PC) and experimental groups, root canals were instrumented up to size F5 using ProTaper Universal rotary instruments (Dentsply Maillefer, Ballaigues, Switzerland). For irrigation, 2 mL of 2.5% sodium hypochlorite was used before each instrument change. Smear layer removal was achieved by final rinsing with 5 mL of 17% ethylenediaminetetraacetic acid for 1 minute, followed by 3 mL of distilled water.

**Fig 1:**
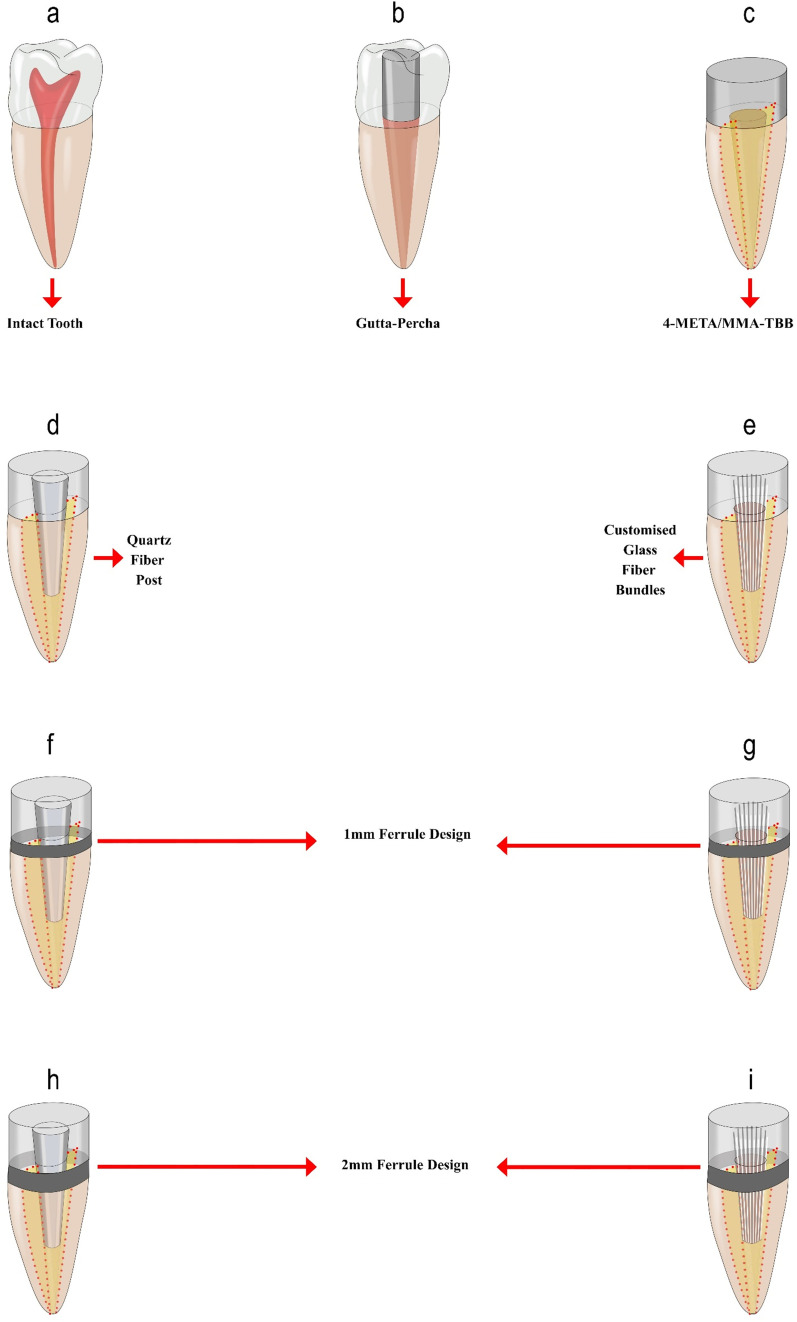
Reattachment designs of the experimental groups. (a) Negative control group (b) Positive control group (c) CB group (d) DT group (e) TB group (f) DT+1F group (g) TB+1F group (h) DT+2F group (i) TB+2F group.

In the experimental groups, root fractures were first generated in the vertical plane in 118 teeth using a hammer and a tapered chisel. The chisel was placed in the center of the root canal, and force was applied using a hammer to induce VRF as described by Wenzel et al. [23]. In total, 48 roots were excluded because of inappropriate or multiple fractures; 70 roots were separated into two equal parts corono-apically and used in the study. The seperated fragments of the roots were measured with a digital caliper and it was ensured that the fragments were two equal parts. The control and experimental groups of this study are listed below and in Fig 1.

### NC group

Specimens were neither instrumented nor obturated.

### PC group

Specimens were instrumented and obturated with a single F5 gutta-percha cone (Dentsply Maillefer, Ballaigues, Switzerland) and AH Plus sealer (Dentsply, DeTrey, Konstanz, Germany). The access cavity was restored with a composite resin material (G-aenial, GC Corporation, Tokyo, Japan).

### CB group

For the reattachment procedures, Superbond C&B (C&B, Sun Medical, Tokyo, Japan) resin cement was used according to the manufacturer’s instructions. The resin cement was applied to the cracked surfaces of the roots. After the application of resin cement, fragments were reattached by using finger pressure and set for 10 minutes. The resin material filled the empty canal and therefore sealed the canal to its terminus. Excess resin was removed with a periodontal curette, and teeth were placed into their individual silicone molds for proper polymerization.

### DT group

Reattachment procedures were performed using Superbond C&B and D.T. Light-Post (Bisco Dental Products, Schaumburg, IL, USA). The halves of the fractured fragments were lightly filled with Superbond C&B resin cement, and the fiber posts were also placed simultaneously (10 mm length). Then, seperated fragments were reattached as described above. Excess resin was removed, and teeth were placed into their individual silicone molds for proper polymerization.

### TB group

Reattachment procedures were performed using Superbond C&B and Tescera fiber bundles (Bisco Dental Products, Schaumburg, IL, USA) in the root canal. The halves of the fractured fragments were lightly filled with Superbond C&B resin cement. The fiber bundles were impregnated with a solvent-free resin (Clearfill SE Bond Primer; Kuraray Medical) for at least 10 minutes, and two layers of impregnated fiber bundles were placed on the root canals of both fragments (10 mm length). Care was taken to ensure that the fibers do not adhere to the side walls of the root canals as this may prevent proper reattachment. Then, seperated fragments were reattached as described above. Excess resin was removed, and teeth were placed into their individual silicone molds for proper polymerization.

### DT+1F group

Reattachment was performed using Superbond C&B and D.T. Light-Post in the root canal and a 1-mm ferrule with Tescera fiber bundles bonded with a Tesceraflo flowable composite (Bisco Dental Products, Schaumburg, IL, USA). Reattachment procedures were applied in the same manner as in the DT group. For the ferrule design, grooves were prepared using depth preparation burs (1 mm depth and 1 mm wide). The grooves were etched, washed, bonded, and light-polymerized for 10 s. Tescera fiber bundles were cut to the length of the groove. The impregnated fiber bundles were placed in the groove and fiber bundles were coated with a Tesceraflo flowable composite, then light-cured for 40 s.

### TB+1F group

Reattachment was performed using Superbond C&B and Tescera fiber bundles in the root canal and a 1-mm ferrule with Tescera fiber bundles with Tesceraflo. Reattachment procedures were applied in the same manner as in the TB group. Ferrule design was performed as previously described for the DT+1F group.

### DT+2F group

Reattachment was performed using Superbond C&B and D.T. Light-Post in the root canal and a 2 mm ferrule with Tescera fiber bundles with Tesceraflo. Reattachment procedures were applied in the same manner as in the DT group. Ferrule design was performed as previously described for the DT+1F group (1 mm depth and 2 mm wide).

### TB+2F group

Reattachment was performed using Superbond C&B and Tescera fiber bundles in the root canal and a 2 mm ferrule with Tescera fiber bundles with Tesceraflo. Reattachment procedures were applied in the same manner as in the TB group. Ferrule design was performed as previously described for the DT+1F group (1 mm depth and 2 mm wide).

In all experimental groups, root canals were instrumented, VRFs were generated, and fragments were reattached using 4-META/MMA-TBB resin-based cement (Superbond C&B) according to the manufacturer’s instructions. Quartz fiber posts (D.T. Light-Post) and/or glass fiber bundles (Tescera) were utilized during reattachment in the DT, TB, DT+1F, TB+1F, DT+2F, and TB+2F groups. For the ferrule design, fiber bundles were covered with a flowable dual composite (Tesceraflo) in the DT+1F, TB+1F, DT+2F, and TB+2F groups. In all experimental groups, core build-ups (4 mm high) were created using a light-cured restorative composite material (G-aenial, GC Corporation, Tokyo, Japan).

Each root was embedded in autopolymerizing acrylic resin with a 0.2-mm layer of silicone impression material to simulate the periodontal ligament. Acrylic blocks were placed on the lower plate of a universal testing machine to which a steel ball (3 mm diameter) was mounted. The tip was lowered for contact with the entire coronal root surface and subjected to a gradually increasing axial force (0.5 mm/min) directed parallel to the long axis of the root. The load necessary to fracture each sample was recorded in Newton (N). After testing the compressive strengths, samples were analyzed with a stereomicroscope with light transillumination (Olympus SZ 6045 TR Zoom stereomicroscope, Olympus Optical Co., Tokyo, Japan) at an original magnification of x40 to determine the fracture type. Fracture types were classified as follows: (1) core fracture (repairable), (2) VRFs with two fragments, (3) fracture with three or more fragments (non-repairable), and (4) fracture at the reattachment line.

Statistical analyses were performed using one-way analysis of variance (ANOVA), and subsequent comparisons among the groups were performed using Duncan’s multiple range tests. Statistical significance was set at p-value <0.05.

## Results

One-way ANOVA and Duncan’s multiple range tests revealed significant differences among the study groups (p < 0.05). Table 1 presents the descriptive statistics of the analyses, initial fracture loads (measured in N), and statistically significant differences. The highest mean initial fracture load was recorded in the NC group, and there was no significant difference between the PC group (p > 0.05). The DT group exhibited significantly lower fracture strength than all the other test and control groups (p < 0.05). The TB+2F group had the highest mean fracture strength among all the experimental groups, however there was no significant difference between the other experimental groups (except the DT group) (p > 0.05). When the ferrule design was performed, it was observed that there was no significant difference between different ferrule designs (1 mm vs. 2 mm) in terms of fracture resistance regardless of the post type (p > 0.05).

**Table 1:** Mean values of the groups in N and descriptive statistics of the analyses.

Group	N	Mean (Newton)	Standard deviation	Standard error of mean	Minimum	Maximum	Median
**NC**	10	1036.70^a^	77.126	24.389	936.60	1195.10	1021.7
**PC**	10	989.66^ab^	43.635	13.799	911.20	1081.40	993.10
**CB**	10	958.84^b^	50.766	16.054	870.40	1045.50	967.65
**DT**	10	871.89^c^	62.297	19.700	791.90	996.50	864.60
**TB**	10	941.82^b^	85.358	26.993	838.70	1087.20	943.35
**DT+1F**	10	951.66^b^	57.465	18.172	846.40	1036.80	963.55
**TB+1F**	10	965.70^b^	49.773	15.739	862.70	1037.60	978.30
**DT+2F**	10	967.10^b^	48.033	15.189	870.40	1029.10	976.80
**TB+2F**	10	981.56^ab^	82.462	26.077	862.10	1086.90	986.10

(Different superscript letters indicate significant differences among the groups; p < 0.05).

**Fig 2:**
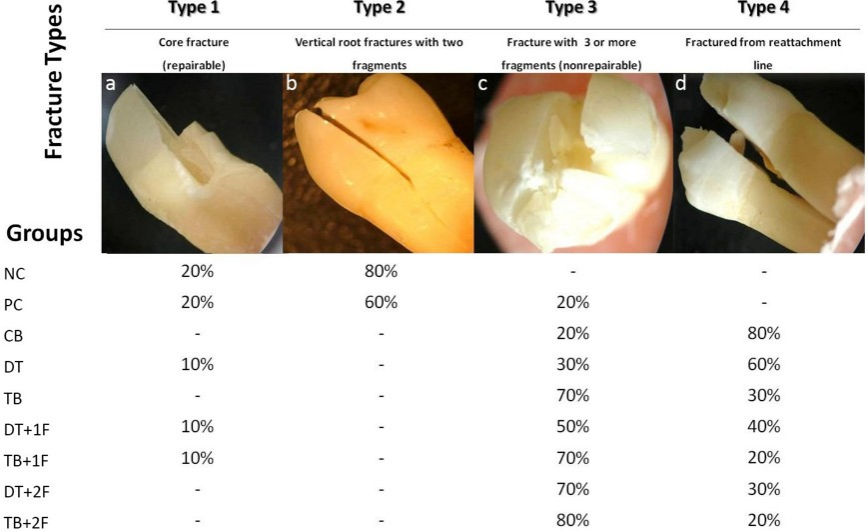
Classification and distribution of fracture types after compressive loading.

Four fracture types occurred following the loading test. Fig 2 represents these fracture types and their distributions. Groups containing specimens reattached using fiber bundles, with or without a ferrule design (TB group, TB+1F group, and TB+2F group), exhibited mainly Type 3 (non-repairable) fractures, while those without fiber bundles and a ferrule design (CB group and DT group) showed fracture at the reattachment line (Type 4).

## Discussion

The first hypothesis of this study was rejected because the reattached fragments with fiber posts and/or customized fiber bundles showed significantly different fracture strengths and fracture types following vertical loading. The second hypothesis was partially accepted, because the ferrule design increased the fracture strength of the reattached teeth with rigid fiber post; however, the ferrule design did not have a significant effect on the fracture strength of the reattached teeth with fiber bundles.

In this study, all controllable factors were standardized. Specimens with similar buccolingual and mesiodistal widths and crown and root dimensions were selected. All roots were instrumented using the same technique, and root fragments were reattached using the same cement. The variable factors comprised of two different fiber materials and seven different application designs.

Extraction was previously considered the only feasible treatment option for vertically fractured teeth [24]. However, many successful reimplantation cases, with reattachment followed up for 1–4 years, have been reported recently [8,15–18,20]. Nizam et al. [19] reported promising clinical findings for the reimplantation of 21 vertically fractured maxillary incisors reattached using 4-META/MMA-TBB. A success rate of approximately 90% was noted at the end of 12 months. In a more recent study, Okaguchi et al. [20] presented the successful clinical outcomes of six complete VRF teeth reattached using 4-META/MMA-TBB resin, concluding that intentional reimplantation combined with root fragment bonding with 4-META/MMA-TBB resin is a successful treatment modality for preserving teeth with VRF.

Fragments in vertical fracture cases have been reattached using self-etch or dual-cured adhesive resins [16,18]. These materials require strict moisture control, and the bonding stage can be cytotoxic [25]. In this study, 4-META/MMA-TBB was selected for reattachment because recent studies demonstrated its superior bonding ability as compared to other cements [26]. For instance, Hayashi et al. [17] and Kudou and Kubota [15] reported successful reattachment when using this cement, and Yıldız et al. [27] obtained higher fracture resistance in endodontically treated roots with VRF.

In this study, a rigid prefabricated quartz fiber D.T. Light-Post with reportedly high fracture strength was selected [28]. However, our findings indicate that rigid posts do not adequately strengthen vertically fractured teeth. Despite the posts exhibiting high fracture strength, reattached teeth showed significantly lower fracture strengths as compared to the control and other test groups. The posts also caused fractures, mainly at the reattachment line. This can be explained by the stiffness of the prefabricated post, as discussed in previous studies [29,30]. Following a comparison of prefabricated and customized fiber posts, the authors reported that prefabricated fiber-reinforced composite (FRC) posts minimized peak stress; however, their stress values were still higher as compared to customized posts. While prefabricated posts more accurately mimicked the natural behavior of a healthy tooth, they also generated more stress, particularly in the dentin. Şen et al. [21], in a reattachment experiment, found that flexible polyethylene fibers are superior to glass fibers due to their composition and fiber thickness and emphasized the negative effect of humidity on glass fiber strength. Customized composite posts were found to yield significantly lower stress near the apex of the post as compared to prefabricated posts, reducing the risk of fracture in regions where conservative clinical interventions were not possible. In another study, Lassila et al. [31] demonstrated that customized FRC posts offer higher flexural strength than prefabricated posts. The authors explained these findings by optimizing the polymer matrix and the properties of the fibers that function as a composite material. Polymethylmethacrylate chains were found to plasticize the cross-linked bisphenol A-glycidyl methacrylate-based matrix of a customized FRC, consequently reducing stress formation at the fiber-matrix interface during deflection [31]. These results show that rigid, prefabricated fiber posts are not preferable for reattachment.

The elastic properties of customized post systems are more suitable than that of rigid fiber posts, and their elasticity values resemble those of a natural tooth [32]. The fiber bundles (Tescera, Bisco) used in this study transferred stress in a more uniform manner across the roots and strengthened the roots to a greater extent than the rigid posts. The fiber bundles resulted in non-repairable multiple fragments (70%), while the D.T. Light-Posts generated fractures at the reattachment line (60%).

According to previous studies, 1–2 mm ferrules created by crowns are preferred because they can increase the fracture resistance of restored teeth and help maintain cement integrity around the restoration [33]. It has also been reported that preparing a tooth with a ferrule supports the mechanical integrity of the restorative elements, regardless of the post or tooth type [34]. However, ferrule created with flexible fibers is not recorded in the dental literature. This study revealed that the ferrule was efficient in strengthening vertically fractured teeth in *ex vivo* conditions, particularly when used with the rigid fiber post.

The mean fracture strength in the TB group (specimens reattached with fiber bundles) was similar to that of the PC group. Therefore, this technique can be considered suitable for vertically fractured teeth. The 2 mm ferrule design yielded the highest mean fracture strength among all the test groups. Ambica et al. [35] recommended the creation of a ferrule using materials with mechanical properties similar to those of dentin, which results in better stress distribution and greater fracture resistance. The results of this study demonstrated that the presence of a ferrule increases the fracture resistance of reattached vertical fragments. This finding is of particular interest because of the current lack of published data regarding these techniques. Ferrules created with crowns have been reported to decrease the compressive strength of dentin at the cervical level and increase tensile stress in the palatal-cervical dentin [36].

Although the fracture strength of reattached vertically fractured teeth is an important factor for clinical success, the difference in the clinical periodontal parameters of the reattached teeth and natural teeth should be considered. Under *in vivo* conditions, it is very important to maintain the viability of periodontal ligament cells. Special attention should be paid during replantation procedures to prepare the groove around the cervical area of the root and apply fiber bundles with adhesives quickly and accurately. If the teeth are held under dry conditions during the reattachment procedure and the extraoral time exceeds 15 minutes, root resorption can occur after reimplantation, threatening the success of the procedure [37]. Therefore, 4-META/MMA-TBB is advantageous as it is not affected by blood contamination and can be polymerized within extraction sockets, eliminating the need for longer polymerization processes seen with dual-cure adhesives [19].

The present study had several limitations. First, the biological aspects of the examined treatments could not be investigated; however, the mechanical aspects related to adhesion, fracture strength, and fracture patterns were compared to those of intact and root canal-treated teeth. As an *ex vivo* study, it did not replicate oral conditions directly, but rather applied a compressive load to test the fracture resistance of restored teeth [38]. Under *in vivo* conditions, most pulpless teeth are likely to fail because of fatigue failure; thus, resistance to static loads should not be the only subject of investigation [39]. Future investigations should use cyclic loading as a more adequate method to reproduce clinically verified fatigue failure [40]. In this study, vertical forces were simulated because the vertically fractured roots were investigated in terms of reattachment behavior. Another limitation of this study was that the teeth were not restored with crowns; hence, testing the post and core preparations did not reflect common clinical practices. However, crown placement following endodontic restoration testing has been questioned, and some studies suggest that crowns can obscure the effects of different post and core build-up techniques [41]. Furthermore, a systematic review reported that there was no obvious difference between the crown and composite in terms of non-catastrophic failures of the restoration or post at 3 years [42]. Therefore, the roots analyzed in this study were restored using fiber materials and a composite core build-up without crown restoration.

## Conclusions

The following conclusions were made:

1. Customized flexible fiber bundles demonstrated significantly higher fracture strength as compared to rigid posts. Although they significantly strengthened the reattached teeth, considerable non-repairable fractures were noted after vertical loading.
2. Rigid fiber posts reduced the fracture strength of reattached roots and resulted in non-repairable fractures after vertical loading. Therefore, rigid fiber posts may not be suitable for reattachment of vertically fractured teeth. However ferrule design may be preferred in case of reattachment with rigid fiber posts.
3. In the treatment of vertically fractured teeth, the novel design of 2 mm ferrule and reattachment using fiber bundles may be preferable to obtain similar fracture strength to the intact teeth. To analyze other clinical situations and define the exact indications or protocols, further studies investigating other factors, such as thermal cycling of the specimens, fatigue loading, and different types of fibers or adhesives, should be planned.

## Supporting information

S1 Dataset Dataset for fracture strength tests and fracture types. (XLSX)
